# Control of blood glucose in type 2 diabetes without weight loss by modification of diet composition

**DOI:** 10.1186/1743-7075-3-16

**Published:** 2006-03-23

**Authors:** Mary C Gannon, Frank Q Nuttall

**Affiliations:** 1 Metabolic Research Laboratory and Section of Endocrinology, Metabolism & Nutrition, VA Medical Center, Minneapolis, MN, USA; 2 Department of Food Science & Nutrition, University of Minnesota, USA; 3 Department of Medicine, University of Minnesota, USA

## Abstract

**Background:**

Over the past several years our research group has taken a systematic, comprehensive approach to determining the effects on body function (hormonal and non-hormonal) of varying the amounts and types of proteins, carbohydrates and fats in the diet. We have been particularly interested in the dietary management of type 2 diabetes. Our objective has been to develop a diet for people with type 2 diabetes that does not require weight loss, oral agents, or insulin, but that still controls the blood glucose concentration. Our overall goal is to enable the person with type 2 diabetes to control their blood glucose by adjustment in the composition rather than the amount of food in their diet.

**Methods:**

This paper is a brief summary and review of our recent diet-related research, and the rationale used in the development of diets that potentially are useful in the treatment of diabetes.

**Results:**

We determined that, of the carbohydrates present in the diet, absorbed glucose is largely responsible for the food-induced increase in blood glucose concentration. We also determined that dietary protein increases insulin secretion and lowers blood glucose. Fat does not significantly affect blood glucose, but can affect insulin secretion and modify the absorption of carbohydrates. Based on these data, we tested the efficacy of diets with various protein:carbohydrate:fat ratios for 5 weeks on blood glucose control in people with untreated type 2 diabetes. The results were compared to those obtained in the same subjects after 5 weeks on a control diet with a protein:carbohydrate:fat ratio of 15:55:30. A 30:40:30 ratio diet resulted in a moderate but significant decrease in 24-hour integrated glucose area and % total glycohemoglobin (%tGHb). A 30:20:50 ratio diet resulted in a 38% decrease in 24-hour glucose area, a reduction in fasting glucose to near normal and a decrease in %tGHb from 9.8% to 7.6%. The response to a 30:30:40 ratio diet was similar.

**Conclusion:**

Altering the diet composition could be a patient-empowering method of improving the hyperglycemia of type 2 diabetes without weight loss or pharmacologic intervention.

## Introduction

Diabetes generally is classified into two large groups, type 1 and type 2. Type 1 is most common in children. In this type of diabetes, the insulin producing beta cells of the pancreas have been destroyed and thus are unable to make insulin. Therefore, the treatment for type 1 diabetes is insulin replacement, without which the individual will die.

Type 2 is most common in adults, indeed, ~95% of people with diabetes have type 2 diabetes [1]. In this type of diabetes, the beta cell mass may be reduced [2,3] but more importantly, there is an impaired ability to make and secrete insulin in response to a rise in glucose concentration. Since people with type 2 diabetes tend to be overweight, weight loss usually is recommended initially. When this fails, oral agents are given. If the latter are not effective, insulin treatment is instituted.

Our long-term objective has been to develop a diet that does not require weight loss, oral agents, or insulin, but still controls blood glucose in people with type 2 diabetes.

In this paper we briefly review data generated in our laboratory regarding the effects of protein, carbohydrate and fat ingestion alone or in mixed meals on circulating glucose and insulin concentrations. These data provided the rationale for the design of several test diets. We refer to these diets as **Lo**w **B**iologically **A**vailable **G**lucose (LoBAG) diets.

Our studies indicate that a decrease in metabolically available dietary glucose, associated with an increase in protein and fat, over an extended period of time, can significantly lower the integrated blood glucose concentration. The decrease is comparable to that obtained using oral agents and occurs without weight loss.

## Background and review

### Rationale for changing the type of carbohydrate in the diet

Carbohydrates are classified as monosaccharides, disaccharides and polysaccharides (Table 1).

**Table 1:** Classification of carbohydrates

**Monosaccharides**	**Disaccharides**	**Polysaccharides**
Glucose	Sucrose	Starch
Fructose	Lactose	Fiber (Non-starch polysaccharides)
Galactose		

The monosaccharides are glucose, fructose and galactose. The disaccharides are sucrose and lactose. They all are commonly referred to as "sugars". The sucrose molecule consists of one molecule of glucose condensed with one molecule of fructose. Thus, sucrose is 50% glucose and 50% fructose. The lactose molecule consists of one molecule of glucose condensed with one molecule of galactose. Thus, lactose is 50% glucose and 50% galactose. The polysaccharides are the starches and fiber. The latter also is referred to as non-starch polysaccharide. Starches are polymers of hundreds to thousands of glucose molecules attached to each other forming a large spherical structure. Thus, starch is 100% glucose. The naturally occurring fiber present in foods, by definition, is not digestible, and has little or no effect on blood glucose or insulin [4,5].

Based on a series of studies, our research group has determined that it is the glucose content of foods from the above carbohydrate sources that is largely responsible for raising blood glucose after meals. Consequently, to reduce the glucose content of the diet, the type of carbohydrate in the foods in the diet should be considered, in addition to the total carbohydrate content.

Starches, as found in cereals, potatoes, rice and pasta, etc. are essentially 100% glucose. Sucrose (table sugar) or its surrogate, a mixture of glucose and fructose, as found in fruits, juices, vegetables, and also high fructose corn syrups, are approximately 50% glucose. Lactose, found in milk products, is 50% glucose. Thus, theoretically, substituting dietary sugars for starches should reduce the post-meal glucose area response by ~50%.

In single meal, single food studies, we have shown that the glucose area response to sucrose or lactose [6] is indeed ~50–60% of the glucose area response to readily digestible starches [7]. In independent studies, we also demonstrated that both fructose [6,8] and galactose [9] modestly increase the plasma insulin concentration even though they have little effect on the glucose concentration.

In order to test whether the glucose area response would be reduced when sugars were substituted for starches in mixed meals, we designed two types of mixed meals, which we called American Meals, and Low Starch Meals [10]. They were very similar in protein, carbohydrate and fat content (Table 2). The major difference between the meals was that the low starch meals contained very little starch.

**Table 2:** Dietary composition of meals

American Meals	Low Starch Meals
20% Protein	22% Protein
40% Carbohydrate	43% Carbohydrate
40% Fat	35% Fat

Three identical meals of each type were given to people with type 2 diabetes at 0800, 1200, 1700, with a snack at 2100 hours. The meals were isocaloric. The glucose and insulin concentrations were determined over a 24-hour period.

**Figure 1:**
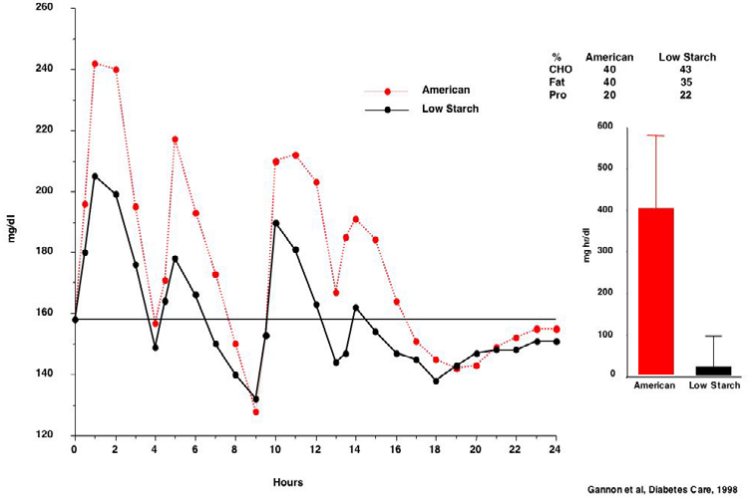
Plasma glucose response in 6 males with untreated diabetes following ingestion of "American meals" (top, broken red line) and "low starch meals" (bottom, solid black line). Bars indicate 24-hour net glucose area response using the fasting glucose concentration as baseline.

When the net area under the curves was determined, using the fasting glucose concentration as baseline, there was essentially no net increase in the blood glucose concentration over the 24-hour period when the low starch meals were ingested (Figure 1).

Thus, we documented that decreasing the dietary starch content in mixed meals, without a change in total dietary carbohydrate, resulted in a major (96%) decrease in the net 24-hour blood glucose area response, i.e. an even greater response than anticipated.

In summary, our data indicate that the metabolic response to carbohydrates depends upon the type of carbohydrate. Readily digestible starches, which are 100% glucose, clearly increase the glucose concentration and increase the insulin concentration. Ingestion of sucrose and/or lactose, which are 50% glucose, results in an increase the plasma glucose which is ~50% less than with starch, and is due largely to the glucose content of these sugars. Sucrose and lactose increase the insulin concentration to a modestly greater extent than expected from the glucose content alone. Overall, in a mixed meal study, substitution of sugars for starches considerably reduced the meal-related increase in plasma glucose over a 24-hour period.

### Rationale for increasing the protein content of the diet

Amino acids derived from protein are converted to glucose through gluconeogenesis. In 1915, Janney reported that 3.5 g of glucose were produced from 6.25 g of ingested meat protein [11]. Thus, theoretically and actually, for every 100 g protein ingested, 56 g of glucose can be produced. For other proteins the range of glucose produced was 50–84 g.

However, in 1924, Dr. MacLean in England gave 250 g meat, which contains ~50 g protein to a subject with type 2 diabetes whose fasting glucose concentration was ~280 mg/dl [12]. Following ingestion of the beef, the glucose concentration remained stable for the 5 hours of the study. When the subject was given 25 g glucose on a separate occasion, the amount of glucose that theoretically could have been produced from the 50 g protein in the 250 g meat, the glucose concentration increased to nearly 600 mg/dl.

**Figure 2:**
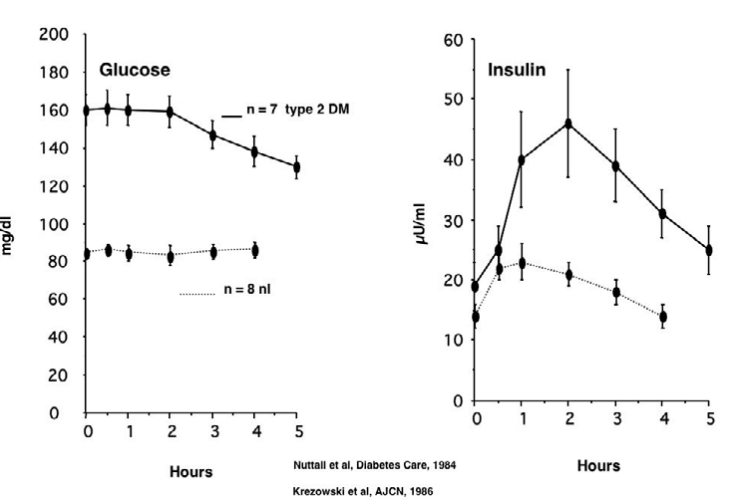
Glucose (left panel) and insulin (right panel) response to 50 g protein, given in the form of very lean beef to 8 normal subjects (bottom, broken lines) and 7 subjects with type 2 diabetes (top, solid lines).

With this [12] and other information [13-18], several years ago, we determined the glucose and insulin responses to 50 g of protein given in the form of lean beef to 8 normal subjects [19] and 7 subjects with type 2 diabetes [20]. When normal subjects ingested the 50 g protein, the plasma glucose concentration remained stable during the 4 hours of the study. When subjects with type 2 diabetes ingested 50 g protein, not only was the glucose stable, it actually decreased (Figure 2).

In normal subjects there was a modest increase in insulin concentration. However, when subjects with type 2 diabetes ingested protein, the insulin concentration increased markedly (Figure 2).

In normal subjects, the insulin increase was only 30% of that to 50 g glucose [19], but in people with type 2 diabetes, it was equal, i.e. 100% [20]. In addition, ingestion of 50 g beef protein had very little effect on glucose production either in normal subjects [21] or in people with type 2 diabetes [22].

The studies cited above were ***single meal ***studies testing the effect of ***dietary protein alone***. From these and other studies we concluded that in people with type 2 diabetes, dietary protein is a potent insulin secretagogue. In addition, protein does not increase blood glucose. Protein actually decreases blood glucose, even though amino acids derived from digestion of the protein can be used for gluconeogenesis. Subsequently we demonstrated that dietary protein acts synergistically with ingested glucose to increase insulin secretion and reduce the blood glucose response to the ingested glucose in people with type 2 diabetes [20,23].

In order to determine the effect of substituting protein for carbohydrate in ***mixed meals ***over an ***extended period of time ***we designed a study in which we increased the protein content of the diet from 15% in the control diet to 30% in the test diet, i.e. we doubled the protein content of the diet [24]. To accommodate the increase in protein, we decreased the carbohydrate content from 55% in the control diet to 40% in the test diet. Since 56 g of glucose could be produced from each 100 g protein ingested [11], the carbohydrate in the diet, plus the glucose produced from the additional protein, would represent a potential carbohydrate content of 48%. The fat content was 30% in both groups. Twelve people with untreated type 2 diabetes were randomized in a crossover design in which they were on each diet for 5 weeks with a washout period in between. The diets were isocaloric, the subjects were weight stable, and all food was provided.

**Figure 3:**
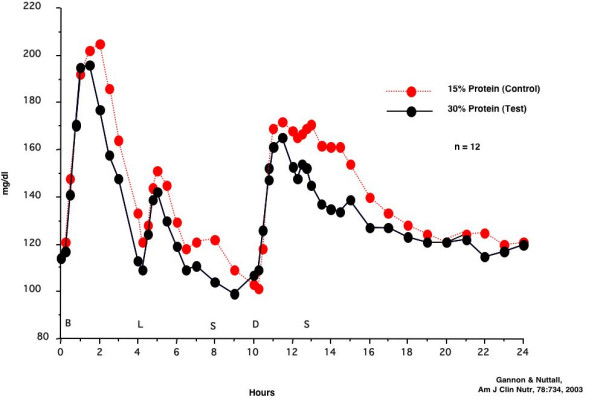
Plasma glucose response in 12 subjects with type 2 diabetes. The response to the control diet (15% protein) is shown in the top, dotted red line. The response to the test diet (30% protein) is shown in the bottom, solid black line).

**Figure 4:**
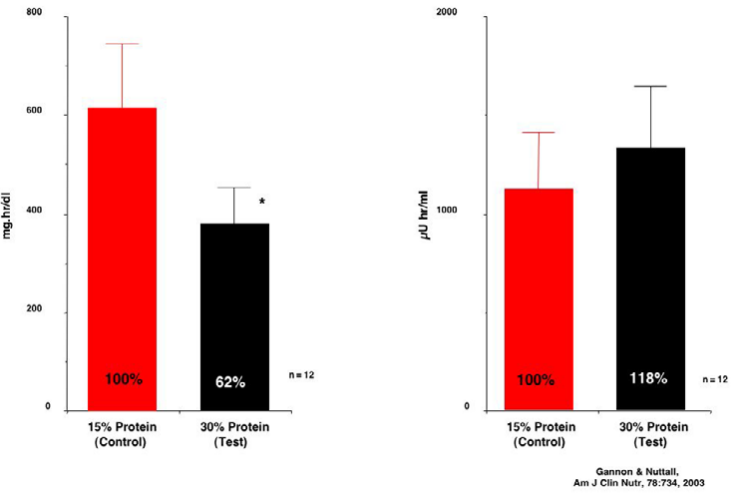
Net 24-hour integrated glucose (left) and insulin area responses (right) to ingestion of a 15% protein (red bar) or 30% protein (black bar) diet in 12 subjects with type 2 diabetes.

The plasma glucose concentrations during the 24-hour period at the end of the 5 weeks on the control diet, or 5 weeks on the high protein diet, are shown in Figure 3. The blood sampling was started at 8 am. Breakfast, lunch, dinner and snack are shown on the X-axis. The differences appear modest. However, when these data are integrated over 24 hours, using the fasting glucose concentration as baseline, the integrated glucose area actually was reduced by 38% on the high protein diet (Figure 4).

In spite of the lower integrated glucose area, the integrated insulin area response was increased by 18% when compared to the control (15% protein) diet results.

**Figure 5:**
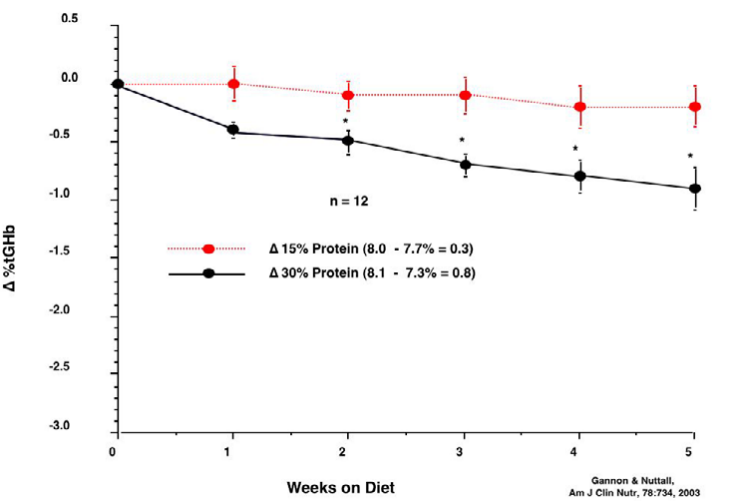
% total glycohemoglobin response to a 15% protein diet, (top, broken red line) and a 30% protein diet (bottom, solid black line) in 12 people with type 2 diabetes.

Most importantly, with the 30% protein diet, the % total glycohemoglobin (%tGHb) decreased from 8.1 to 7.3 (Δ = 0.8) (Figure 5). It decreased from 8.0 to 7.7% during the control (15% protein) diet (Δ = 0.3). The difference was statistically significant by week 2.

In summary, increasing dietary protein from 15% to 30% of total food energy at the expense of carbohydrate resulted in an increased integrated insulin concentration, a decreased 24 hour integrated glucose concentration, and a decreased %tGHb.

These data were presented in 2004 at the Kingsbrook Conference on Nutritional and Metabolic Aspects of Low Carbohydrate Diets [25], and an adaptation of that presentation was later published [26].

### Rationale for increasing the fat content of the diet

As shown by us (unpublished data), and others [27], when ingested independently, fats do not effect the circulating blood glucose concentration. Fats can delay the digestion and/or absorption of dietary carbohydrates, but this appears be fat-source specific [unpublished data]. Also, when ingested with a carbohydrate-containing food, fats can decrease the glucose concentration, and/or increase the insulin concentration [28]. This effect is likely to be fat-source dependent as well [unpublished data].

**Figure 6:**
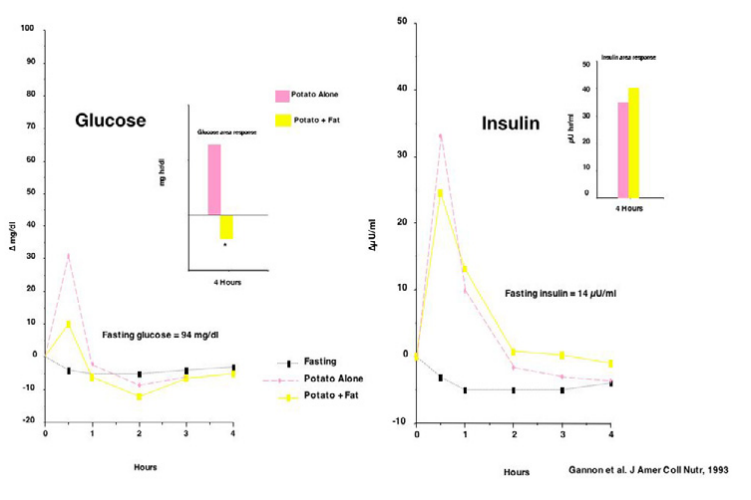
Glucose (left panel) and insulin (right panel) responses to ingestion of 50 g fat alone, or 50 g fat + 50 g carbohydrate in normal young subjects.

An example of fat decreasing the blood glucose concentration is shown in Figure 6. In this study, normal young subjects were given 50 g carbohydrate in the form of potato, with or without 50 g butter [29]. When the subjects ingested butter with potato, the 4 hour integrated glucose area was actually negative, whereas it was strongly positive with potato alone. Even though the glucose area response was reduced by butter, the integrated insulin area was approximately the same [29]. The results were similar to those obtained by Collier and associates earlier [30]. The data were of great interest to us since they suggested that dietary fat, or at least butter, may be important in lowering the blood glucose in people with diabetes. Therefore, we did the same study in people with type 2 diabetes.

**Figure 7:**
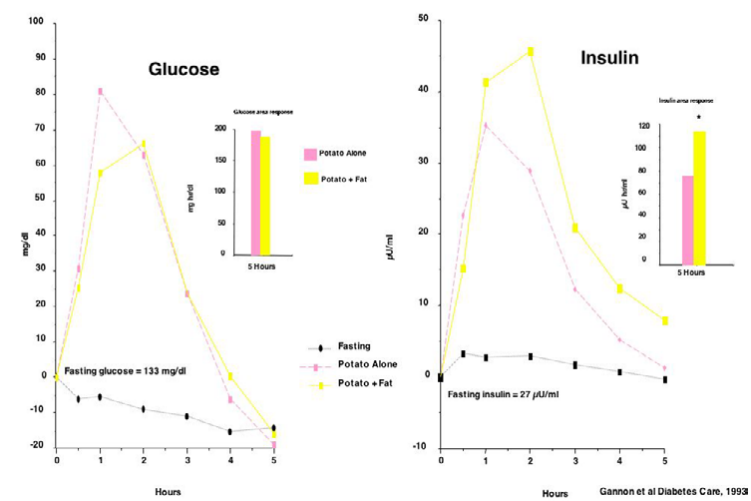
Glucose (left panel) and insulin (right panel) responses to ingestion of 50 g fat alone, or 50 g fat + 50 g carbohydrate in subjects with type 2 diabetes.

When the same study was done in older people with type 2 diabetes, there was essentially no effect on blood glucose by ingestion of butter with the potato when compared to the effect of potato ingested alone [31] (Figure 7). This was in contrast to the striking reduction in glucose response noted in non-diabetic young subjects. However, the addition of butter clearly stimulated an increase in insulin concentration in people with type 2 diabetes. The reason for the differences is unknown.

In summary, ongoing studies in our laboratory suggest that the effect of fat on plasma glucose and insulin responses is likely to be complex, and fat-source dependent. In any regard, substituting dietary fat for carbohydrate should result in a reduction in post-meal glucose rise in people with type 2 diabetes, which may or may not be due merely to a smaller amount of carbohydrate in the diet. Also, these results indicated to us that data obtained in normal subjects cannot always translate to subjects with type 2 diabetes.

### Integrating principles from previous studies

Our previous studies have allowed identification of several principles regarding macronutrients and blood glucose response in people with type 2 diabetes. In our most recent studies, these principles have been integrated into a comprehensive approach to the dietary management of type 2 diabetes. Specifically, the principles involved are 1) a decrease in total carbohydrate content, 2) a decrease the metabolically available glucose content of the diet by changing the composition of the carbohydrate in the diet, i.e. decreasing the starch, and 3) an increase in the protein content of the diet. All may contribute to an improvement in blood glucose concentration. Basically, a combination of these incorporated into a diet we refer to as **Lo**w **B**iologically **A**vailable **G**lucose Diets (LoBAG diets).

Based on the above principles, we have designed a diet in which the carbohydrate was decreased from 55% to 20%. The protein was increased from 15% to 30%. The fat was increased from 30% to 50%, keeping the saturated fat constant at ~11%. We refer to this as a LoBAG_20_, diet with the subscript of 20 added because the diet contains 20% total carbohydrate. Eight men with untreated type 2 diabetes were studied using a randomized crossover design [32]. Subjects were on each diet for 5 weeks with a washout period in between. The diets were isocaloric; subjects remained weight stable. All food was provided.

**Figure 8:**
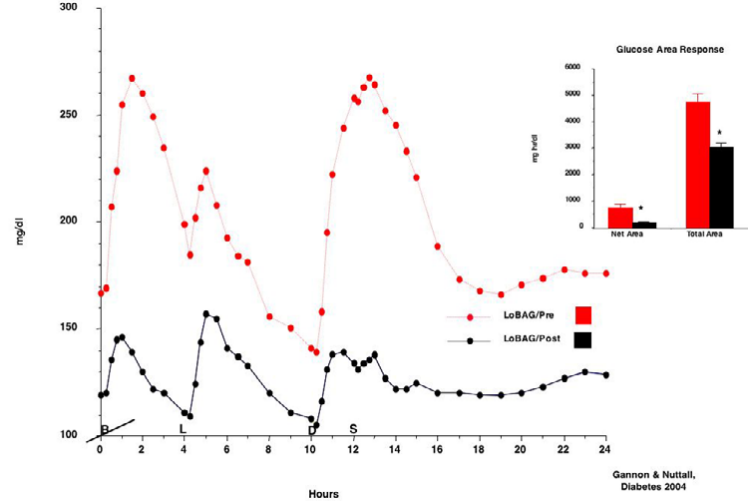
Plasma glucose response before (top broken red line) and after 5 weeks on a LoBAG_20 _diet (bottom solid black line) in 8 men with untreated type 2 diabetes. Net area (left set of bars), using the fasting glucose concentration as baseline and total area (right set of bars), using zero glucose as baseline, before (red bars) and after 5 weeks on the diet (black bars).

The postprandial glucose concentrations were markedly attenuated following 5 weeks on the LoBAG_20 _diet (Figure 8). In addition, the fasting glucose concentration was significantly decreased as well. The net area response, using the fasting concentration as baseline was significantly decreased (by 65%) on the LoBAG_20 _diet. Similarly, the total area response, using zero as baseline, was decreased by 45%.

**Figure 9:**
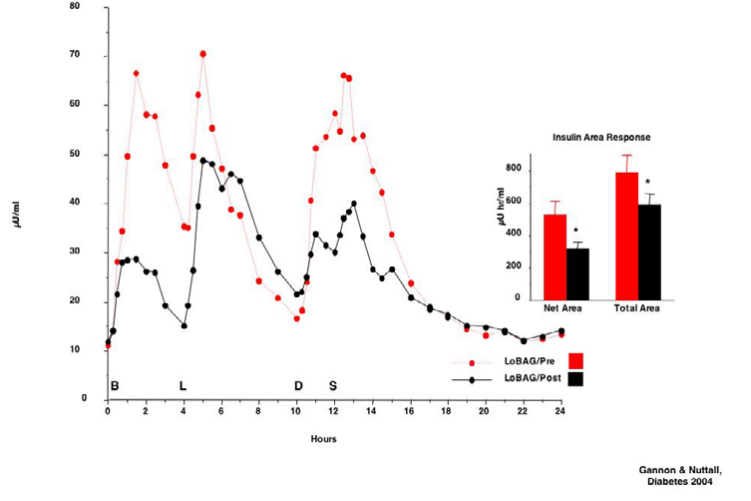
Serum insulin response before (top broken red line) and after 5 weeks on a LoBAG_20 _diet (bottom solid black line) in 8 men with untreated type 2 diabetes. Net area (left set of bars), using the fasting insulin concentration as baseline and total area (right set of bars), using zero insulin as baseline, before (red bars) and after 5 weeks on the diet (black bars).

The fasting serum insulin concentrations were similar, but the excursions after meals were significantly greater when ingesting the standard diet, i.e. before the LoBAG_20 _diet (Figure 9). The net insulin area response, using the fasting insulin concentration as baseline, was decreased by 40%. The total insulin area response, using zero as baseline, was decreased by 25% following 5 weeks on the LoBAG_20 _diet. Both insulin area decreases were statistically significant.

**Figure 10:**
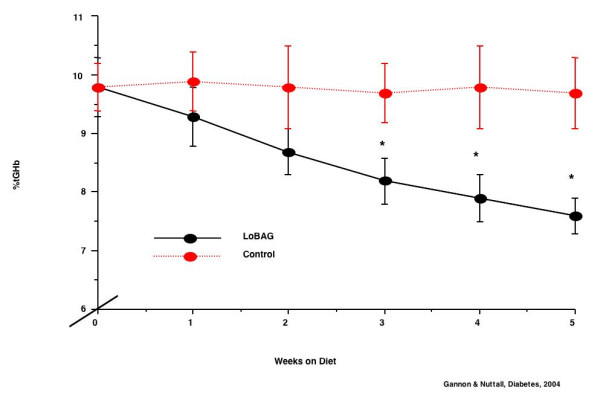
% total glycohemoglobin during the 5 weeks on the control diet (top, broken red line) and during the 5 weeks on the LoBAG_20 _diet (bottom, solid black line).

The %tGHb on the control diet remained stable during the 5 weeks of the study (Figure 10). However, when on the LoBAG_20 _diet, the %tGHb decreased continually during the five weeks, and was still decreasing linearly at the end of the study. The difference was statistically significant by week 3.

**Figure 11:**
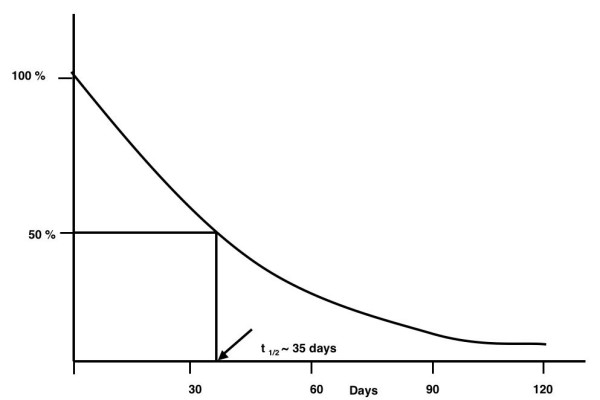
Theoretical rate of change of % total glycohemoglobin following an instantaneous change in blood glucose concentration.

The time required for the %tGHb to reflect a new steady-state glucose concentration is essentially the time required for the turnover of the red blood cell mass, which is 100–120 days (Figure 11). However, the time required for a 50% change is only 35 days [33]. That is why our studies were conducted for 5 weeks, or 35 days.

On the control diet, the glycohemoglobin was 9.8% and remained unchanged (Table 3). On the LoBAG_20 _diet, the glycohemoglobin decreased from 9.8% to 7.6% over the 5 weeks of the study. Extrapolating the data to ~120 days (~15 weeks), theoretically, the glycohemoglobin would be 5.4%, which is within the normal range.

**Table 3:** Change in % total glycohemoglobin

Diet	Pre	Post (5 weeks)	Extrapolated (~15 weeks)
Control	9.8%	9.8%	9.8%
LoBAG_20_	9.8%	7.6%	5.4%

Reference range = 4.0 – 6.3%

### Future studies

We are completing a study in which the LoBAG_20 _diet has been modified by increasing the carbohydrate content to 30% from 20% with a corresponding decrease in fat. We refer to this as a LoBAG_30 _diet. This was done because the diet is likely to be acceptable to a greater number of people. The results to date appear similar to those obtained when the subjects received the LoBAG_20 _diet.

### Summary

Overall, our data indicate that it is possible to improve blood glucose control in people with type 2 diabetes by relatively simple adjustments in diet, and without weight loss.

It should be understood that we consider these studies to be proof of concept studies. Larger studies involving both men and women, and for longer periods of time will be required in order to determine the applicability of this approach to the treatment of diabetes. In addition, further modifications may improve the results and/or make the dietary change more acceptable.

## Competing interests

The author(s) declare that they have no competing interests.

## Authors' contributions

Both authors were equally responsible for designing the experiments, evaluating the statistics, interpreting the data, writing the manuscript, and organizing the figures and tables.
